# A Targeted, Low-Throughput Compound Screen in a *Drosophila* Model of Neurofibromatosis Type 1 Identifies Simvastatin and BMS-204352 as Potential Therapies for Autism Spectrum Disorder (ASD)

**DOI:** 10.1523/ENEURO.0461-22.2023

**Published:** 2023-05-15

**Authors:** Alex Dyson, Megan Ryan, Shruti Garg, D. Gareth Evans, Richard A. Baines

**Affiliations:** 1 Division of Evolution, Infection and Genomics, School of Biological Sciences, Faculty of Biology, Medicine and Health, University of Manchester, Manchester Academic Health Science Centre, Manchester M13 9PT, United Kingdom; 2 Division of Neuroscience, School of Biological Sciences, Faculty of Biology, Medicine and Health, University of Manchester, Manchester Academic Health Science Centre, Manchester M13 9PT, United Kingdom; 3 Child and Adolescent Mental Health Services, Royal Manchester Children’s Hospital, Central Manchester University Hospitals NHS Foundation Trust, Manchester Academic Health Sciences Centre, Manchester M13 9WL, United Kingdom

**Keywords:** autism spectrum disorder, *Drosophila*, drug screening, neurofibromatosis type 1, Nf1

## Abstract

Autism spectrum disorder (ASD) is a common neurodevelopmental condition for which there are no pharmacological therapies that effectively target its core symptomatology. Animal models of syndromic forms of ASD, such as neurofibromatosis type 1, may be of use in screening for such treatments. *Drosophila* larvae lacking *Nf1* expression exhibit tactile hypersensitivity following mechanical stimulation, proposed to mirror the sensory sensitivity issues comprising part of the ASD diagnostic criteria. Such behavior is associated with synaptic dysfunction at the neuromuscular junction (NMJ). Both phenotypes may thus provide tractable outputs with which to screen for potential ASD therapies. In this study, we demonstrate that, while loss of *Nf1* expression within the embryo is sufficient to impair NMJ synaptic transmission in the larva, constitutive *Nf1* knock-down is required to induce tactile hypersensitivity, suggesting that a compound must be administered throughout development to rescue this behavior. With such a feeding regime, we identify two compounds from a targeted, low-throughput screen that significantly and consistently reduce, but do not fully rescue, tactile hypersensitivity in *Nf1^P1^* larvae. These are the HMG CoA-reductase inhibitor simvastatin, and the BK_Ca_ channel activator BMS-204352. At the NMJ, both compounds induce a significant reduction in the enhanced spontaneous transmission frequency of *Nf1^P1^* larvae, though again not to the level of vehicle-treated controls. However, both compounds fully rescue the increased quantal size of *Nf1^P1^* mutants, with simvastatin also fully rescuing their reduced quantal content. Thus, the further study of both compounds as potential ASD interventions is warranted.

## Significance Statement

No therapies currently exist that consistently and effectively target the core symptoms of autism spectrum disorder (ASD), which include altered responses to sensory stimuli. Previously it was shown that *Drosophila* larvae lacking expression of ASD-associated *Nf1* display a heightened response to a mechanical stimulus and increased neuronal excitability, likely because of excessive Ras activity. Here, out of a screen for compounds targeting such mechanisms, we identified simvastatin and BMS-204352 to reduce the likelihood of a response in *Nf1*^−/−^ larvae following mechanical stimulation. These compounds also improved synaptic transmission defects at the neuromuscular junction (NMJ). Such findings support the further study of these drugs as potential ASD therapies in the clinic.

## Introduction

Autism spectrum disorder (ASD) is a common neurodevelopmental condition affecting 1–2% of children (Baird et al., 2006; Maenner et al., 2020). Clinically, it is characterized by impairments in social communication alongside repetitive and restricted behavior and interests, which include altered responses to sensory stimuli (American Psychiatric Association, 2013). Such sensory impairments may directly contribute to other ASD symptoms and correlate with worsening outcomes on several quality-of-life measures. Thus, they may provide an important target for therapeutic intervention (Lundqvist, 2015; Robertson and Baron-Cohen, 2017; Lin and Huang, 2019). The need for such interventions is urgent, given the lifelong impact on an individual’s quality of life that ASD can impose (Lord et al., 2020), and the substantial economic burden arising from the need to support those affected (Buescher et al., 2014; Leigh and Du, 2015). Currently, the only two compounds approved by the Food and Drug Administration for the treatment of behavioral symptoms in ASD are the atypical antipsychotics aripiprazole and risperidone, both of which are prescribed for irritability and aggression (Lord et al., 2020). Additional medications commonly prescribed to individuals with ASD include stimulant drugs for the treatment of comorbid ADHD, selective serotonin-reuptake inhibitors for mood disturbances and anxiety, and melatonin for sleep disorders (Aishworiya et al., 2022). However, these are often associated with adverse side effects ranging from the mild (e.g., weight gain, appetite changes, fatigue, and headaches) to the severe (e.g., metabolic syndrome; Aishworiya et al., 2022). Importantly, none have been shown to consistently and effectively improve abnormal sensory sensitivity or indeed other aspects of core ASD symptomatology (Hyman et al., 2020).

While ∼75% of ASD cases are idiopathic, ∼4–5% occur in association with a monogenic neurodevelopmental syndrome (Fernandez and Scherer, 2017). Because syndromic forms of ASD have a known single causative mutation, they are comparatively simpler and offer a more tractable model from a biomedical research perspective (Sztainberg and Zoghbi, 2016). One such condition is neurofibromatosis type 1, an autosomal dominant disorder arising from loss of function of the *NF1* gene on chromosome 17 (Gutmann et al., 2017). The prevalence of ASD among individuals with neurofibromatosis type 1 is estimated at 10–25%, with up to a further ∼20% exhibiting some form of clinically relevant symptomatology (Garg et al., 2013; Walsh et al., 2013; Adviento et al., 2014; Plasschaert et al., 2015; Morris et al., 2016; Eijk et al., 2018). Furthermore, it is becoming increasingly apparent that ASD and neurofibromatosis type 1 share an overlapping pathophysiology. Thus, animal models of neurofibromatosis type 1 possess significant potential in screening for novel therapies for ASD (Molosh and Shekhar, 2018; Kaczorowski et al., 2020).

An effective drug screen foremost requires a suitable model of the disorder. Target-based, *in vitro* approaches have not proven suitable for conditions like ASD in which the underlying disease biochemistry is poorly understood and likely involves the dysfunction of multiple pathways. They also do not routinely permit behavioral outputs as measures of drug efficacy (Pandey and Nichols, 2011; Strange, 2016). More traditional, preclinical model organisms, such as mice, are equally unfeasible, given their high cost of maintenance, long generation time, and relatively small litters (Bell et al., 2009). By contrast, the fruit fly, *Drosophila melanogaster*, overcomes many of these limitations and provides a useful bridge between the two model systems (Bell et al., 2009; Pandey and Nichols, 2011; Strange, 2016). The potential of *Drosophila* in neurodevelopmental drug discovery was exemplified by a screen of 2000 small molecules for their ability to rescue glutamate-induced lethality in a fly model of ASD-associated Fragile X syndrome (Chang et al., 2008). Indeed, identification of GABA as a hit compound lent support for the use of GABA-promoting drugs in clinical trials (Braat and Kooy, 2015), although the success of these has been mixed (Berry-Kravis et al., 2012; Erickson et al., 2013, 2014; Ligsay et al., 2017; Veenstra-VanderWeele et al., 2017).

*Drosophila* express a highly conserved ortholog of *NF1* (The et al., 1997) with similar molecular, cellular, and behavioral functions to its mammalian counterpart (Guo et al., 2000; Walker et al., 2006). Accordingly, *Nf1*^−/−^ flies display phenotypes analogous to ASD in the clinic, including altered communication (Moscato et al., 2020), disrupted sleep (Bai and Sehgal, 2015), and repetitive behaviors in the form of excessive grooming (King et al., 2016, 2020). More recently, it was shown that *Drosophila* larvae lacking *Nf1* expression exhibit an increased likelihood of responding to a mechanical stimulus, thought to mirror the sensory sensitivity abnormalities comprising part of the ASD diagnostic criteria (Dyson et al., 2022). Here, we exploit this phenotype in a targeted, low-throughput screen to identify compounds that improve tactile hypersensitivity in *Nf1^P1^* larvae. Any such leads may therefore have potential in managing ASD symptomatology in affected individuals. We start by investigating the temporal requirements for *Nf1*, to determine when compounds must be administered for optimal activity. Then, using a protocol of feeding drugs during both embryonic and larval stages, we identify two compounds, simvastatin and BMS-204352, as capable of consistently improving, but not fully rescuing, the enhanced response to mechanical touch. Finally, we demonstrate that both compounds also reduce *Nf1*-associated synaptic transmission deficits at the neuromuscular junction (NMJ).

## Materials and Methods

### Fly lines and maintenance

The *Nf1^P1^* mutant and its parental K33 line used in this study (The et al., 1997) were initially obtained from Seth Tomchik (University of Iowa), where they were both backcrossed into the *w^CS10^* background such that K33 acts as an isogenic control, as described previously (King et al., 2016; Dyson et al., 2022). For the temperature-dependent knock-down of *Nf1* at different developmental stages, *elav^c155^-GAL4;tubP-GAL80^ts^* was crossed to either *UAS-Nf1^RNAi^;UAS-Dicer2* (experiment) or *UAS-GFP^RNAi^;UAS-Dicer2* (control). These lines were generated by combining the following constructs together: *elav^c155^-GAL4* (Bloomington, #458; Giachello and Baines, 2015), *tubP-GAL80^ts^* (McGuire et al., 2004), *UAS-Nf1^RNAi^* (VDRC ID #109637; King et al., 2016), *UAS-GFP^RNAi^* (Bloomington #9331; Roignant et al., 2003), and *UAS-Dicer2* (Dietzl et al., 2007). In these experiments, animals were maintained at either 30°C to permit GAL4-dependent expression of *UAS-Nf1^RNAi^* and thus knock-down of endogenous *Nf1*, or at 18°C to facilitate GAL80-dependent repression of GAL4, thereby allowing the expression of endogenous *Nf1* (McGuire et al., 2004). Progenitor flies were set up to mate and left to deposit embryos on grape agar plates, which were collected at 4-h intervals before being transferred to food-containing vials. For the constitutive knock-down of *Nf1*, embryos and larvae were maintained constantly at 30°C until the experiment. For the embryonic knock-down of *Nf1*, embryos were maintained at 30°C for 17–21 h after egg laying (AEL), and then transferred to 18°C (Ashburner et al., 2005). For the larval knock-down of *Nf1*, embryos were maintained at 18°C for 42- to 46-h AEL, and then transferred to 30°C (Ashburner et al., 2005). For all other experiments, lines were maintained at 25°C. Fly food was standard cornmeal medium. All flies were kept in a 12/12 h light/dark cycle.

### Compound administration

Compounds that were tested for their ability to improve tactile hypersensitivity in *Nf1^P1^* larvae are listed in Table 1. All compounds were made up as stock solutions in DMSO and stirred into molten fly food cooled to ≤60°C. Typically, progenitor flies were maintained on food containing either compound or vehicle (DMSO) for ≥3 d before being transferred onto fresh compound-containing or vehicle-containing food, from which larvae for the experiment were collected. However, for experiments examining embryonic exposure to compound, progenitor flies were maintained on food containing either compound or vehicle (DMSO) for ≥3 d before being transferred onto standard food (lacking compound), from which larvae for the experiment were collected. For experiments examining larval exposure to compound, progenitor flies were maintained on standard food before being transferred onto fresh compound-containing or vehicle-containing food, from which larvae for the experiment were collected. DMSO concentration was 0.05% for all experiments presented in this study.

**Table 1:** List of compounds screened for their ability to improve tactile hypersensitivity in *Nf1^P1^* larvae

Process targeted	Compound	Mechanism of action	Source
Ras/MAPK signalling	Lovastatin	HMG CoA-reductase inhibitor	Generon
	Fluvastatin sodium		Generon
	Simvastatin		Fluorochem
	Atorvastatin calcium		Generon
	Binimetinib	MEK inhibitor	Generon
	U0126		Cambridge Bioscience
	GDC-0973 (Cobimetinib)		Generon
	PD 0325901		Merck
	Salirasib	Ras inhibitor	Generon
	GNE 3511	DLK inhibitor	Generon
	Palbociclib HCl	CDK4/6 inhibitor	Generon
	Vorinostat	HDAC inhibitor	Generon
	Belinostat		Generon
	Trichostatin A		Cambridge Bioscience
	BAY293	Ras/SOS1 interaction inhibitor	Generon
Ion channel function	ML 6733	K_2P_ activator	Generon
	BMS-204352	BK_Ca_ channel activator	Cambridge Bioscience/Scientific Laboratory Supplies
	BAY K 8644	L-type Ca_v_ channel activator	Stratech Scientific Ltd
	Lamotrigine	Sodium channel blocker	Fisher Scientific UK Ltd
	SKA 31	SK channel activator	Bio-Techne

Compounds were selected based on either their ability to inhibit Ras/MAPK activity or to modulate ion channel function, mostly with the expectation of reducing neuronal excitability. As part of the “Ras/MAPK” targeting group, inhibitors of histone deacetylase (HDAC) and CDK4/6 were included since they have been demonstrated to improve viability in *Drosophila* models of other RASopathies (Das et al., 2021).

### Tactile sensitivity and compound screening

To assess tactile sensitivity, a mechanical stimulus was applied briefly to the posterior end of each wall-climbing third instar larva (of either sex), as described previously (Dyson et al., 2022). Larvae were counted as “responding” only if they exhibited a full, 360° rolling motion. Video footage exemplifying the typical response of *Nf1^P1^* larvae (representative of larvae lacking *Nf1* expression) compared with that of K33 controls has already been published (Dyson et al., 2022). All such experiments were conducted at room temperature by an experimenter blinded to genotype and/or treatment. For the initial screen, all compounds were tested at 50 μm on *Nf1^P1^* larvae and compared against *Nf1^P1^* larvae raised on an equivalent concentration of DMSO vehicle (*n *=* *20 per treatment group). Data for each compound were accepted if vehicle-treated *Nf1^P1^* larvae exhibited the expected significant increase in likelihood of nocifensive response compared with vehicle-treated K33 larvae (*n *=* *20). For the compound screen, data were normalized with the equation: (no. of compound-treated responders/no. of vehicle-treated *Nf1^P1^* responders) × 100, such that the number of *Nf1^P1^* larvae responding to the stimulus = 100%. Compounds found to exert a significant effect in the initial screen were then tested in four independent validation trials (*n *=* *20 per treatment group per trial, thus *n *=* *80 overall) at 50 μm. For compound validation, % responding larvae per trial [i.e., (no. of responders/20) × 100] for each genotype was calculated.

### Larval crawling

A 77-cm^2^ arena formed of 2% agarose (depth ∼2 mm) within the lid of a clear 96-well plate was placed into a DanioVision observation chamber. A 2- to 3-mm moat was maintained between the agar and the edge of the lid, which was filled with 5 m NaCl to deter larvae from crawling off the agar. Wall-climbing third instar larvae (of either sex) were rinsed in ddH_2_O, then placed onto the center of the arena, and left to acclimatize for 30 s. EthoVision XT Video Tracking Software (part of DanioVision) was used to measure total distance traveled over a 3-min period under white light. Experiments were conducted at room temperature by an experimenter blinded to genotype and/or treatment. Only larvae that remained on the agar for the entire recording period were included in our analyses.

### Electrophysiology

Wall-climbing third instar larvae (of either sex) were fillet-dissected to permit the recording and analysis of excitatory junction potentials (EJPs) and miniature EJPs (mEJPs) in HL3 saline + 1.5 mm CaCl_2_ (Stewart et al., 1994), as described previously (Dyson et al., 2022). EJP amplitude and resting membrane potential were calculated using Clampfit 10.3 (Molecular Devices), while mEJP amplitude and frequency were calculated using MiniAnalysis (Synaptosoft Inc.). Amplitudes were corrected using the equation v’ = E(ln[E/(E-v)]), where v’ refers to the corrected amplitude, v is the recorded amplitude, and E is the driving force, assumed to be equal to resting membrane potential if the reversal potential is 0 mV (Feeney et al., 1998). Quantal content was calculated as corrected EJP amplitude/corrected mEJP amplitude. All recordings were conducted blind to genotype or treatment.

### Statistical analysis

All statistical analyses were conducted using GraphPad Prism (version 8). Pairs of quantitative datasets were compared via a two-tailed, unpaired Student’s *t* test, while more than or equal to three quantitative datasets were analyzed via either one-way (ungrouped) or two-way (grouped) ANOVA, followed by Tukey’s *post hoc* test. Fisher’s exact test was used to compare two sets of categorical variables (e.g., vehicle- vs compound-treated *Nf1^P1^* larvae). All analysis was conducted on raw data before normalization. When analyzing compound validation experiments (see above, Tactile sensitivity and compound screening), comparisons were made between the percentage values of larvae responding per trial, such that sample size is the number of trials (i.e., *n *=* *4). Statistically significant *p* values are given in the figures, while nonsignificant *p* values that are nevertheless relevant to interpreting the data are given in the figure legend.

## Results

### Constitutive knock-down of *Nf1* is required to induce tactile hypersensitivity, while knock-down of *Nf1* in the embryo alone is sufficient to impair synaptic transmission

Because neurofibromatosis type 1 and ASD are primarily developmental disorders, it is probable that they arise, at least partially, from aberrations in brain development and/or function during early life stages that lead to permanent changes in postembryonic behavior (Yenkoyan et al., 2017; Courchesne et al., 2019). These aberrations may be irreversible, and, consequently, less susceptible to clinical intervention in later life (de la Torre-Ubieta et al., 2016). To account for this, we first investigated when the NF1 protein is required during the *Drosophila* life cycle to regulate larval tactile sensitivity. We also focused attention to synaptic transmission at the NMJ, since deficits in the latter may be associated with the hypersensitivity phenotype (Dyson et al., 2022). We used the UAS/GAL4/GAL80^ts^ system to restrict *Nf1* expression to either embryonic or larval stages (McGuire et al., 2004). In these experiments, larvae in which *Nf1* was knocked down via RNA interference were compared with a control line in which a dsRNA construct against GFP was expressed instead.

**Figure 1.**
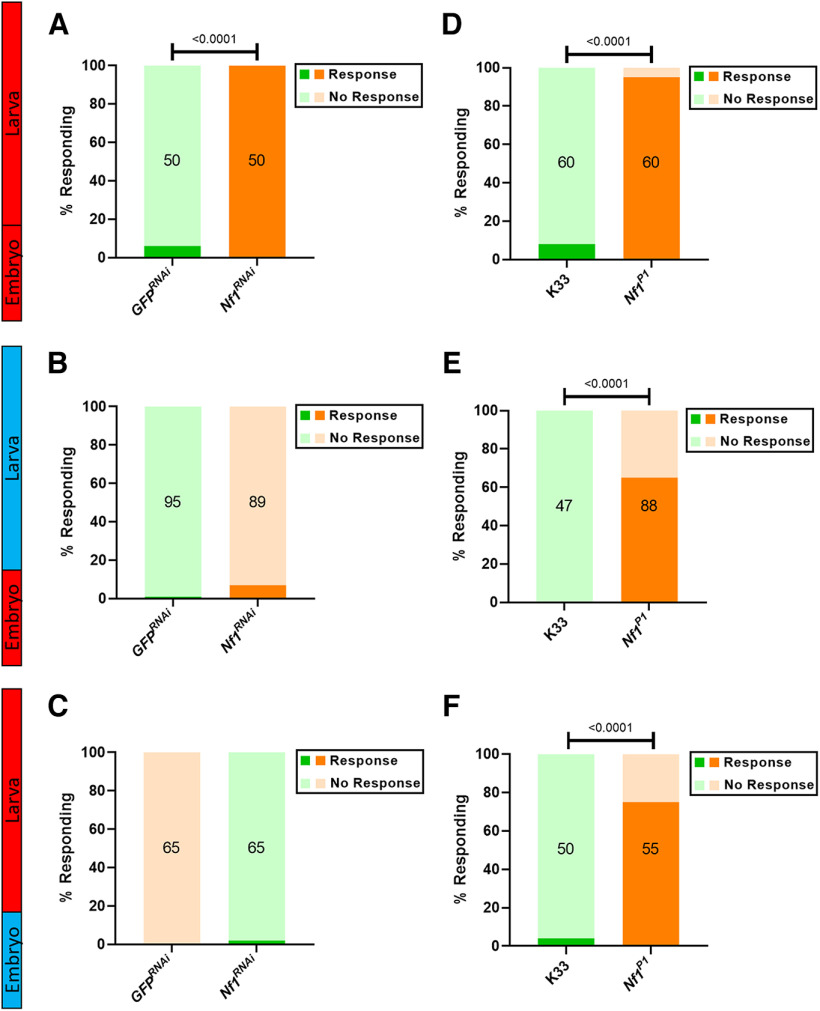
Constitutive knock-down of *Nf1* is required to induce tactile hypersensitivity in third instar larvae. Abbreviated genotypes for the lines tested are given in the figure panels. *GFP^RNAi^* (green) and *Nf1^RNAi^* (orange) refer to lines in which GAL4 is expressed under the control of *elav* to drive expression of either UAS-*GFP^RNAi^* or UAS*-Nf1^RNAi^*, respectively, and UAS-*Dicer2*, with GAL80^ts^ expressed under the control of the *tubulin* promoter. All transgenic constructs are hemizygous or heterozygous in the larvae tested. ***A***, Constitutive knock-down of *Nf1* expression (*Nf1^RNAi^*) throughout all life stages results in larval tactile hypersensitivity, as indicated by a significantly greater number of larvae responding to a mechanical stimulus compared with *GFP^RNAi^*. ***B***, Knock-down of *Nf1* only within the embryo has no significant impact on the number of responding larvae (*p* = 0.0580), nor does (***C***) knock-down of *Nf1* within the larval stages (*p* > 0.9999). ***D–F***, K33 and *Nf1^P1^* larvae were also subjected to the same shifts in temperature as those required for constitutive, embryonic, and larval knock-down of *Nf1*, respectively. Regardless of the temperature paradigm, *Nf1^P1^* larvae always demonstrated a significantly greater likelihood of displaying the nocifensive response following stimulation. Numbers within each bar represent the sample size for that group. For the ease of comparing groups in which different sample sizes were used, raw data have been presented as percentages within the figure. Statistical comparisons via Fisher’s exact test were nonetheless conducted on raw data before normalization.

As expected, constitutive knock-down of *Nf1* resulted in a significant increase in the likelihood of a larva exhibiting a nocifensive response following a brief, typically innocuous, mechanical stimulation (Fig. 1*A*). However, this was not observed when *Nf1* knock-down was restricted solely to the embryonic period (Fig. 1*B*) or, alternatively, to postembryonic larval stages (Fig. 1*C*). To completely rule out the possibility that some nonspecific effect of raising larvae at 18°C, independent of GAL80^ts^ expression, diminishes the nocifensive response, we also exposed *Nf1^P1^* mutant larvae and the isogenic K33 controls to the same shifts in temperature. In all such controls, the number of *Nf1^P1^* larvae displaying the nocifensive response was significantly greater than that of control larvae (Fig. 1*D–F*). Thus, we conclude that the NF1 protein is involved in regulating normal tactile sensitivity during both embryogenesis and larval development.

**Figure 2.**
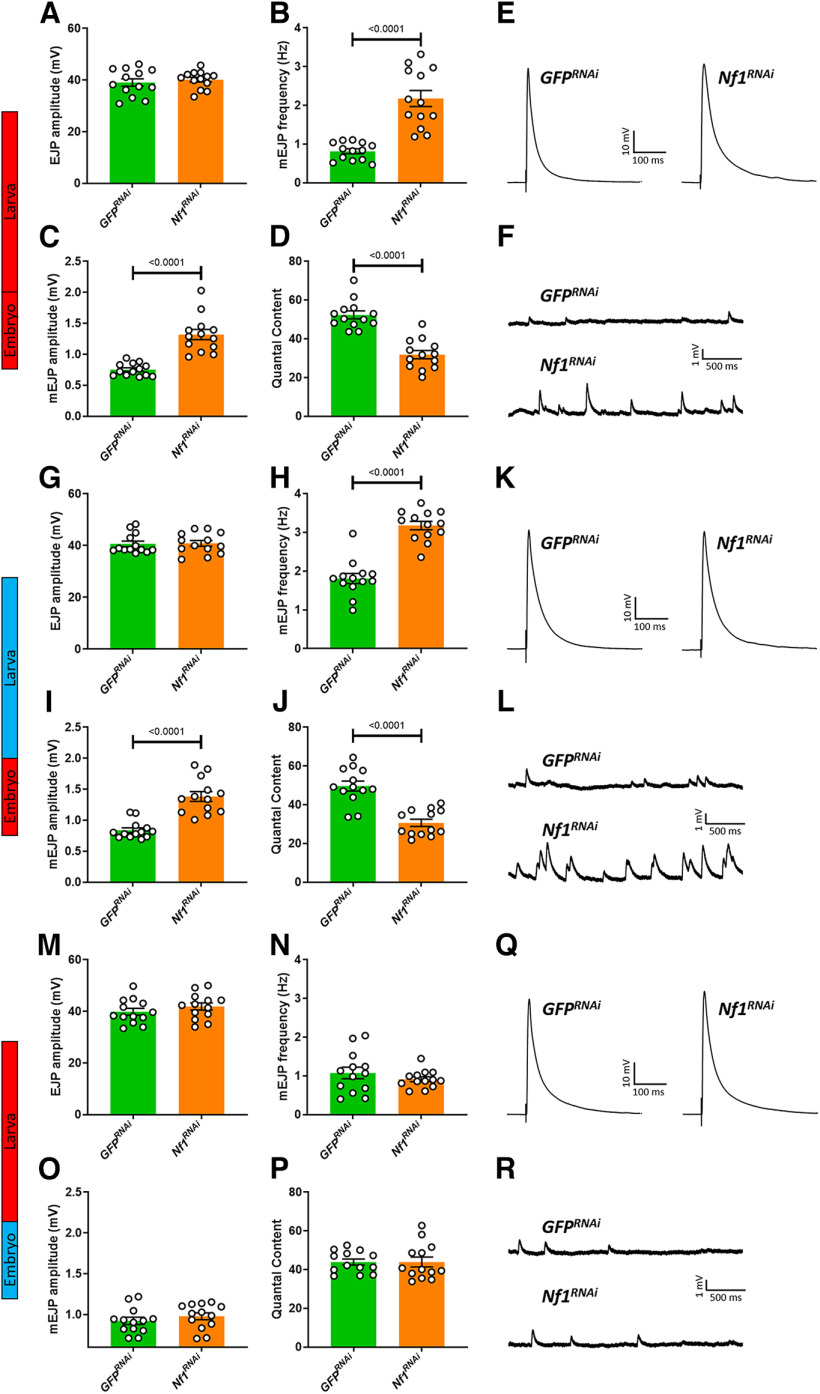
Knock-down of *Nf1* in the embryo is sufficient to induce synaptic transmission deficits in the third instar larval stage. Full genotypes for those abbreviated in the figure (i.e., *GFP^RNAi^* and *Nf1^RNAi^*) are explained in the legend for Figure 1. ***A–F***, Raising *Nf1^RNAi^* larvae at 30°C, sufficient to ensure constitutive knock-down of *Nf1*, mimics the *Nf1*^−/−^ larval phenotype (Dyson et al., 2022), in that it does not affect EJP amplitude (*p* = 0.5334) but increases mEJP frequency and amplitude, and reduces quantal content. ***G–L***, Knock-down of *Nf1* within the embryonic period has a similar effect on synaptic transmission as constitutive knock-down on all parameters examined, including no significant impact on (***G***) EJP amplitude (*p* = 0.8967). ***M–R***, In contrast, knock-down of *Nf1* only during larval stages has no effect on (***M***) EJP amplitude (*p* = 0.3010), (***N***) mEJP frequency (*p* = 0.3230), (***O***) mEJP amplitude (*p* = 0.3694), or (***P***) quantal content (*p* = 0.9998). Data in each panel were analyzed via unpaired, two-tailed Student’s *t* test. For each experiment, *n = *13. Data are presented as mean ± SEM.

Next, we examined the effect of identical shifts in *Nf1* expression on synaptic transmission at the NMJ. In *Nf1*^−/−^ mutants, the frequency of spontaneous transmission events (mEJPs) is significantly increased, while evoked release (quantal content) is significantly reduced. This change is seemingly compensated for by an increase in postsynaptic input resistance, as evidenced via an enhanced quantal size (mEJP amplitude), rendering the amplitude of evoked events (EJPs) unchanged (Dyson et al., 2022). These changes are similarly observed following constitutive *Nf1* knock-down (Fig. 2*A–F*), as well as when knock-down of *Nf1* is restricted to the embryonic period (Fig. 2*G–L*). However, knock-down of *Nf1* during larval stages is insufficient to disrupt synaptic transmission (Fig. 2*M–R*). Together, these data indicate an early developmental requirement for *Nf1* in regulating synaptic transmission at the larval NMJ, but that the role of *Nf1* in sensory sensitivity requires expression at both embryonic and larval stages.

### A targeted screen identifies two compounds sufficient to improve tactile hypersensitivity in *Nf1^P1^* larvae

To expedite the screening process, we opted to assay compounds for their ability to improve tactile hypersensitivity, rather than aberrant synaptic transmission. As knock-down of *Nf1* in both the embryonic and larval periods is necessary to induce this behavioral phenotype, it is plausible that, for a compound to rescue such behavior, it must likewise be present throughout both stages. To achieve this, progenitor flies were provided compound-containing food for ≥3 d before being transferred onto fresh food containing the same compound on which eggs would be laid and larvae would develop. This was to ensure that the compound was present both during the embryonic and larval stages, as feeding compounds to mated females results in significant transfer to embryos (Marley and Baines, 2011). Tested compounds fell broadly into one of two classes of activity (Table 1; Das et al., 2021). First, compounds were selected for their ability to diminish Ras/MAPK signaling, since the NF1 protein acts as a negative regulator of Ras (Ratner and Miller, 2015), knock-down of either *Ras85D* or *Ras64B* fully rescues both tactile hypersensitivity and synaptic dysfunction in *Nf1^P1^* larvae (Dyson et al., 2022), and genetic variants associated with Ras/MAPK pathways are recurrent in idiopathic ASD (Luo et al., 2018). The second class comprised compounds known to modulate ion channel function, typically in favor of reducing neuronal excitability, as synaptic transmission deficits associated with tactile hypersensitivity in *Nf1^P1^* larvae are indicative of neuronal hyperexcitability (Dyson et al., 2022), and an imbalance in central excitatory/inhibitory activity has been proposed as a major pathophysiological mechanism underlying ASD (Rubenstein and Merzenich, 2003).

**Figure 3.**
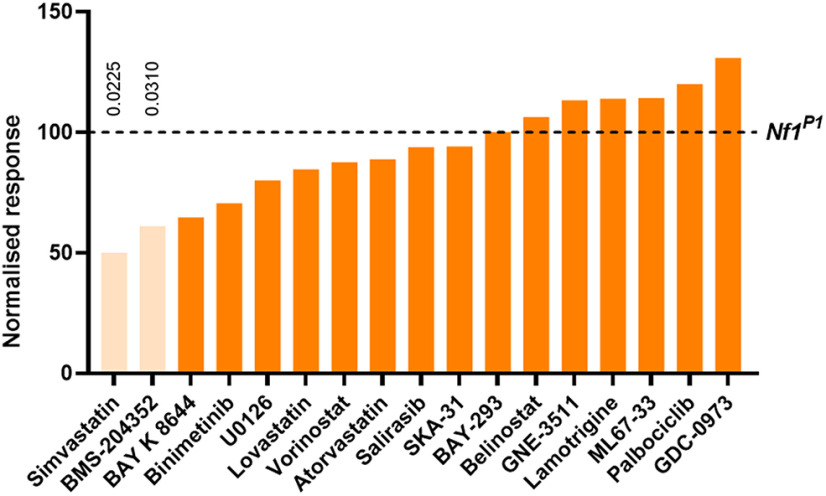
A targeted pharmacological screen identifies simvastatin and BMS-204352 to improve tactile hypersensitivity in *Nf1^P1^* larvae. Twenty compounds were screened for their ability to reduce the number of *Nf1^P1^* larvae responding to a mechanical stimulus, with three proving toxic at the concentration (50 μm) tested. Only administration of simvastatin (50% of vehicle-treated *Nf1^P1^*) and BMS-204352 (61.1% of vehicle-treated *Nf1^P1^*) resulted in a significant decrease in the number of responding larvae. Data are presented as a percentage of the number of *Nf1^P1^* larvae raised on an equivalent concentration of DMSO (vehicle) that responded to the stimulus (dotted line = 100%). Statistical comparisons were conducted on raw data using Fisher’s exact test between compound-treated and vehicle-treated *Nf1^P1^* larvae.

**Figure 4.**
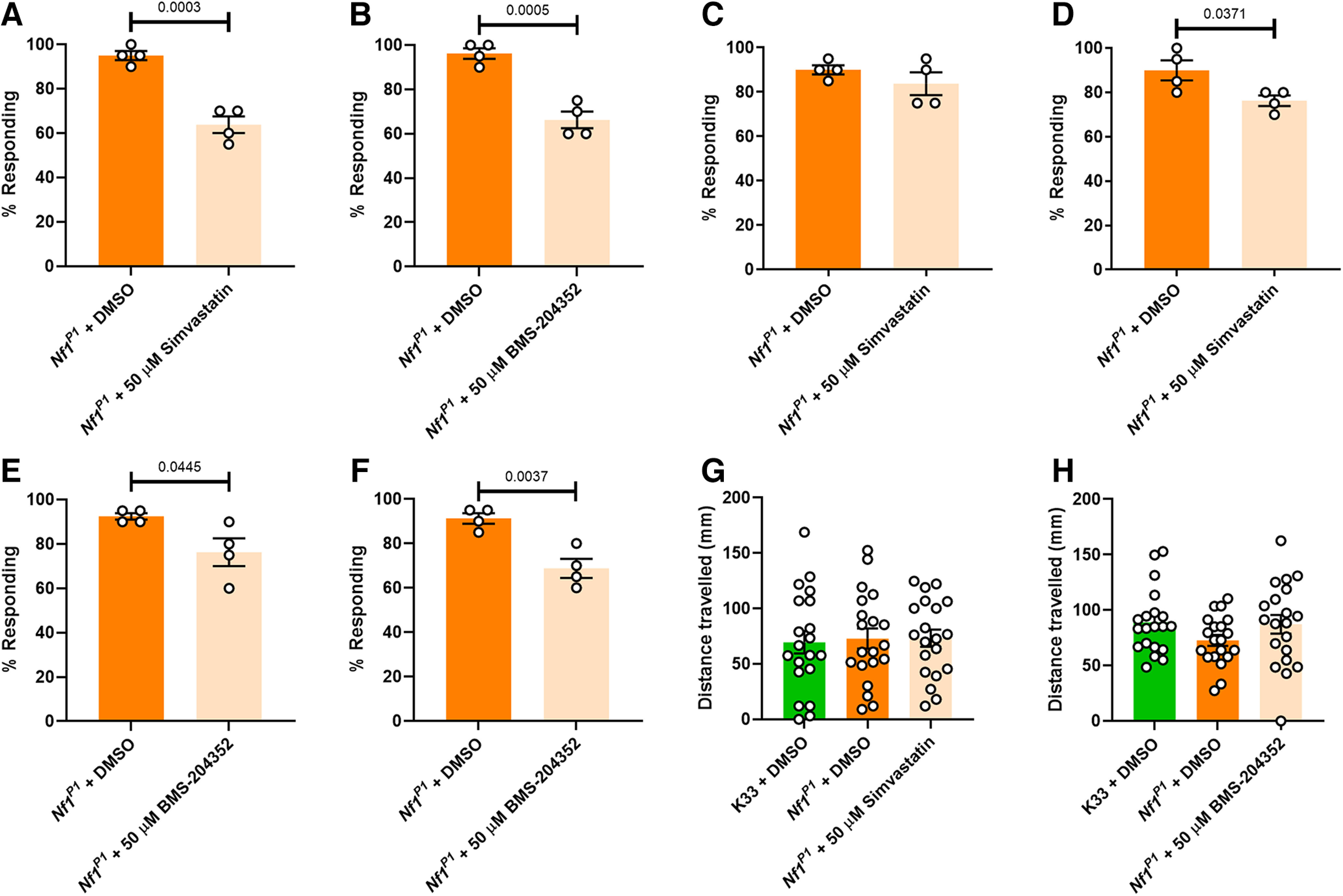
Simvastatin and BMS-204352 consistently improve, but do not fully rescue, tactile hypersensitivity in *Nf1^P1^* larvae, while having no effect on overall activity. ***A***, Across four independent trials, 50 μm Simvastatin significantly reduces the mean percentage responding larvae, as does (***B***) 50 μm BMS-204352. ***C***, The presence of simvastatin solely during the embryo has no impact on the percentage of *Nf1^P1^* larvae responding to stimulation (*p* = 0.3026). ***D***, Conversely, exposing only larval progeny to simvastatin results in a significant reduction in the mean percentage of responding *Nf1^P1^* larvae. ***E***, The presence of BMS-204352 solely during the embryo results in a significant reduction in the percentage of *Nf1^P1^* larvae responding to stimulation. ***F***, The percentage of responding *Nf1^P1^* larvae is also significantly reduced when only larval progeny are exposed to BMS-204352. ***G***, K33 and *Nf1^P1^* larvae do not significantly differ in their total distance traveled over a 3-min period at room temperature, nor is this impacted by treatment with 50 μm simvastatin (*p* = 0.9518). ***H***, 50 μm BMS-204352 treatment also does not alter distance traveled in *Nf1^P1^* larvae, which again show no significant difference in crawling behavior compared with K33 controls (*p* = 0.1881). In panels ***A–F***, each data point represents the percentage responding larvae from a single trial, with *n *=* *20 per trial, such that *n *=* *80 larvae overall. Data in these panels were analyzed via unpaired, two-tailed Student’s *t* test. Comparisons were made between individual trials, such that *n *=* *4 per group. Data in panels ***G***, ***H*** were analyzed via one-way ANOVA followed by Tukey’s *post hoc* test. All data are presented as mean ± SEM.

All drugs were tested at 50 μm (i.e., the concentration added to fly food), although the concentration reaching the CNS is unknown. While three of the compounds tested proved toxic at this concentration (Fluvastatin, PD0325901, and Trichostatin A), we identified two compounds that significantly reduced the number of *Nf1^P1^* larvae responding to mechanical stimulation (Fig. 3). These were the HMG CoA-reductase inhibitor simvastatin, and the big conductance calcium-activated potassium channel (BK_Ca_) activator BMS-204352. Four subsequent independent trials demonstrated that the effect of each compound was consistent, with a significantly lower mean percentage of *Nf1^P1^* responders in the compound-treated versus vehicle-treated groups (Fig. 4*A*,*B*). It is worth noting, however, that each compound exerted a significant effect in only three out of four trials when comparing treatment groups within the trial itself (Table 2).

**Table 2:** Outcomes of four independent trials to validate the efficacy of simvastatin and BMS-204352 following the initial compound screen

Compound (concentration)	Trial no. (post-initial screen)	% Vehicle-treated responders (*n*/20)	% Compound-treated responders (*n*/20)	*p* value
Simvastatin (50 μm)	1	90 (18/20)	55 (11/20)	0.0310
	2	95 (19/20)	70 (14/20)	0.0915 (ns)
	3	100 (20/20)	70 (14/20)	0.0202
	4	95 (19/20)	60 (12/20)	0.0197
BMS-204352 (50 μm)	1	95 (19/20)	60 (12/20)	0.0197
	2	90 (18/20)	60 (12/20)	0.0648 (ns)
	3	100 (20/20)	75 (15/20)	0.0471
	4	100 (20/20)	70 (14/20)	0.0202

The data in the table refer to that depicted in Figure 4*A,B*. For each trial, the number of vehicle-treated and compound-treated *Nf1^P1^* larvae responding to the stimulus is given, both as a fraction of total larvae tested (*n *=* *20 per trial) and as a percentage. The outcome (*p* value) of the statistical comparison between these two groups in each trial is also stated. Both compounds reduced the number of responding larvae in all four validation trials, with this reduction reaching significance (*p* < 0.05) in three out of four trials for each. For trials in which the reduction was not significant, *p* values were nevertheless approaching 0.05. Statistical analysis (via Fisher’s exact text) was conducted on raw data rather than percentage values. ns = not significant.

In addition, we also tested our hypothesis that compound exposure is required in both the embryo and larval stage to improve sensory behavior. Accordingly, administering simvastatin solely during the embryo had no significant effect on the number of responding *NF1^P1^* larvae (Fig. 4*C*). By contrast, raising larvae on simvastatin, without embryonic exposure, significantly reduced the likelihood of a nocifensive response (Fig. 4*D*), but to a lesser degree than when exposing both embryo and the larva (Fig. 4*A*). These observations are consistent with our assumption that, based on *Nf1* knock-down data (Fig. 1), a compound must be present during both embryonic and larval stages if it is to exert its optimal effect. However, in contrast to simvastatin, BMS-204352 administration during the embryo or larvae alone was sufficient to significantly reduce the mean percentage of responding larvae (Fig. 4*E*,*F*), although the effect was stronger with larval exposure. Indeed, administering BMS-204352 solely to larvae appears to be similarly effective as administering the drug throughout both stages (Fig. 4*B*), with changes from 91.3 ± 2.4% to 68.8 ± 4.3% responding (Fig. 4*F*), and 96.3 ± 2.4% to 66.3 ± 3.8% responding (Fig. 4*A*), respectively. Possibly, targeting a mechanism that may occur downstream of excessive Ras signaling, such as disrupted BK_Ca_ activity, is a more robust way of improving behavior, such that pharmacological manipulation is still feasible in the larva.

While additional concentrations (10, 25, and 100 μm) of the two hit compounds were also tested, we found 50 μm the most consistently effective (data not shown). Furthermore, we also considered the possibility that one or both compounds reduce the likelihood of a nocifensive response in *Nf1^P1^* larvae via a nonspecific, sedative effect. To test this, the impact of each compound (50 μm) on larval mobility was measured over a 3-min period. We observed no significant difference in the mean distance traveled by *Nf1^P1^* and K33 larvae (Fig. 4*G*,*H*), nor was distance influenced in *Nf1^P1^* larvae by either simvastatin (Fig. 4*G*) or BMS-204352 (Fig. 4*H*). This supports the effect of these compounds being specific to tactile hypersensitivity, and not because of a global reduction in activity.

### Simvastatin and BMS-204352 improve synaptic transmission deficits at the *NF1^P1^* larval NMJ

**Figure 5.**
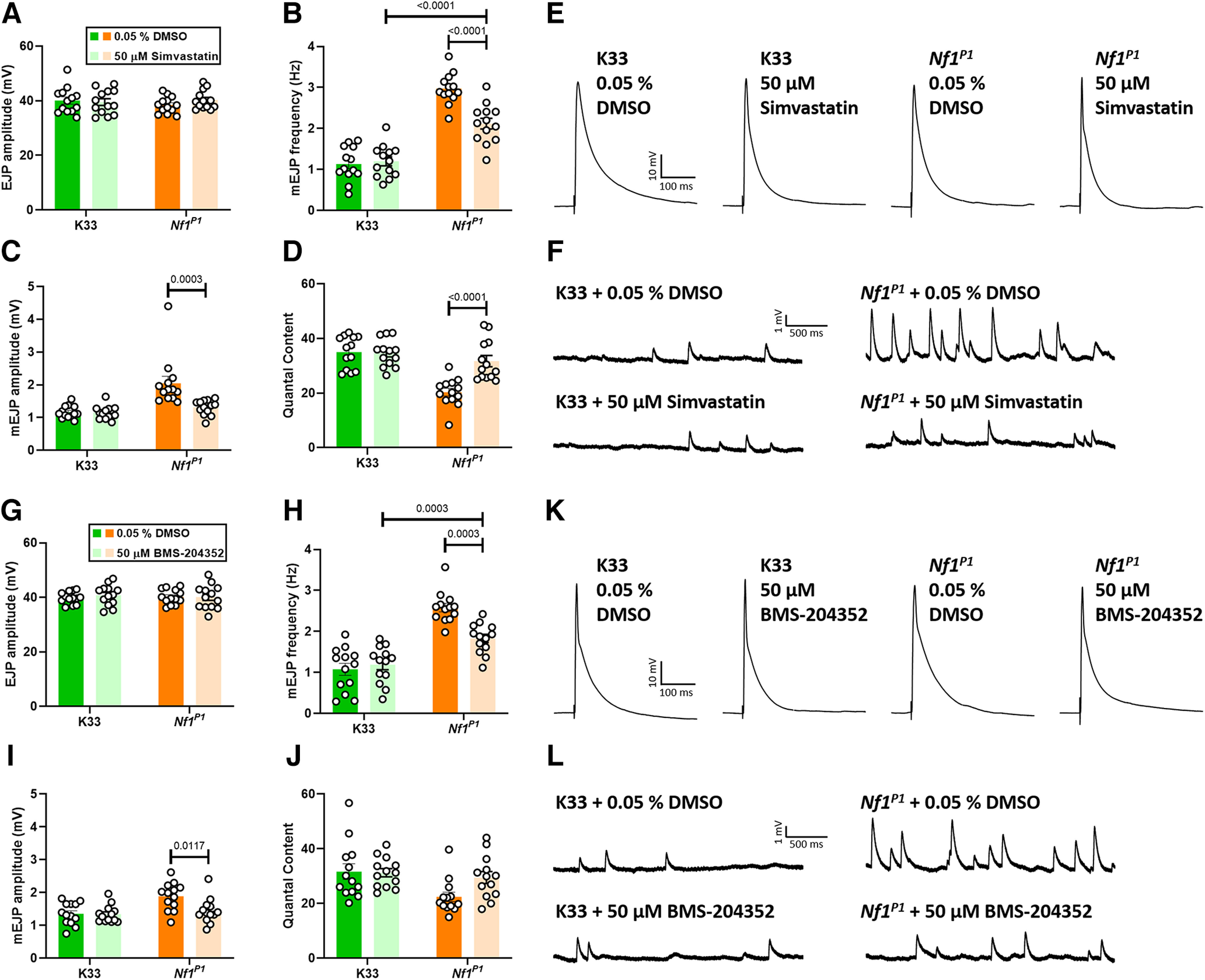
Simvastatin and BMS-204352 improve synaptic transmission deficits at the *Nf1^P1^* larval NMJ, and have no impact on normal transmission in K33 larvae. ***A***, *S*imvastatin (50 μm) treatment has no effect on EJP amplitude in either *Nf1^P1^* or K33 larvae (*p* = 0.2793). ***B***, The increased mEJP frequency of *Nf1^P1^* larvae is reduced following simvastatin treatment, although this is still significantly greater than that of vehicle-treated K33 larvae. There is no significant difference between vehicle-treated and simvastatin-treated K33 larvae (*p* = 0.9784). ***C***, Simvastatin rescues the enhanced mEJP amplitude of *Nf1^P1^* larvae to values indistinguishable from those of vehicle-treated K33 larvae (*p* = 0.8097), which do not show a significant difference compared with simvastatin-treated K33 larvae (*p* > 0.9999). ***D***, Simvastatin rescues the reduced quantal content of *Nf1^P1^* larvae to values indistinguishable from those of vehicle-treated K33 larvae (*p* = 0.4934), which also do not show a significant difference compared with simvastatin-treated K33 larvae (*p* = 0.9989). ***E***, ***F***, Representative traces of data presented in panels ***A–D***. ***G***, BMS-204352 (50 μm) treatment has no effect on EJP amplitude in either *Nf1^P1^* or K33 larvae (*p* = 0.5878). ***H***, The increase in mEJP frequency of *Nf1^P1^* larvae is reduced following BMS-204352 treatment, although this is still significantly greater than that of vehicle-treated K33 larvae. There is no significant difference between vehicle-treated and simvastatin-treated K33 larvae (*p* = 0.9098). ***I***, BMS-204352 rescues the enhanced mEJP amplitude of *Nf1^P1^* larvae to values indistinguishable from those of vehicle-treated K33 larvae (*p* = 0.9211), which do not show a significant difference compared with BMS-204352-treated K33 larvae (*p* = 0.9996). ***J***, The increase in quantal content in *Nf1^P1^* larvae following BMS-204352 treatment is not significant (*p* = 0.1075); however, the quantal content of BMS-204352 treated *Nf1^P1^* larvae also does not differ from that of vehicle-treated K33 larvae (*p* = 0.8849). BMS-204352 does not significantly alter quantal content in K33 larvae either (*p* = 0.9991). All statistical comparisons were made via two-way ANOVA followed by Tukey’s *post hoc* test, in which each genotype + treatment group was compared with all others. *n *=* *13 for each group. All data are presented as mean ± SEM. Although not explicitly stated in the figure or legend, in panels ***B–D*** and ***H–J***, mEJP frequency and amplitude were both significantly increased, and quantal content significantly reduced, in vehicle-treated *Nf1^P1^* larvae relative to vehicle-treated K33 controls, as would be expected in larvae lacking *Nf1* expression (Dyson et al., 2022).

Tactile hypersensitivity in *Nf1*^−/−^ larvae is associated with impaired synaptic transmission at the NMJ, with both phenotypes arising in a Ras-dependent manner (Dyson et al., 2022). Therefore, we next investigated the effect of simvastatin (Fig. 5*A–F*) and BMS-204352 (Fig. 5*G–L*) exposure throughout embryonic and larval stages on activity at this peripheral glutamatergic synapse. Exposure to simvastatin (50 μm) led to a significant reduction in the typically enhanced mEJP frequency of *Nf1^P1^* larvae, although rescue was only partial since this was still significantly greater than that of vehicle-treated K33 controls (Fig. 5*B*). Conversely, the same treatment led to full rescue of the increased quantal size (mEJP amplitude) and reduced quantal content (Fig. 5*C*,*D*). There was no effect on EJP amplitude, which is also unaltered by the *Nf1^P1^* mutation (Fig. 5*A*), and simvastatin treatment had no effect on any parameter measured in K33 larvae. Similar effects on EJP amplitude (Fig. 5*G*), mEJP frequency (Fig. 5*H*), and mEJP amplitude (Fig. 5*I*) were also observed following BMS-204352 (50 μm) treatment, with the exception that the increase in quantal content in *Nf1^P1^* larvae treated with BMS-204352, compared with those treated with DMSO, was not significant (Fig. 5*J*). However, the quantal content of *Nf1^P1^* larvae treated with BMS-204352 was also not significantly different from that of vehicle-treated K33 larvae. Again, there was no effect of BMS-204352 on any parameter measured in K33 larvae.

## Discussion

Clinical trials have thus far failed to identify treatments that effectively and consistently help to manage core ASD symptomatology, including abnormal responses to sensory stimuli. To address this, we examined whether compounds that target either Ras/MAPK signaling or ion channel activity can improve tactile hypersensitivity in *Nf1^P1^* larvae. We also investigated the temporal requirements for *Nf1* in regulating the two ASD-associated phenotypes employed in this study. This is because the onset of certain ASD symptoms may occur as a result of specific developmental disruption; consequently, the time during which treatment is administered may be of equal importance as the molecular mechanism targeted. For example, in a mouse model of the syndromic ASD Tuberous Sclerosis, a four-week regimen of rapamycin treatment beginning at postnatal day 7 prevented the occurrence of impaired social behavior, even four weeks after the end of treatment (Gibson et al., 2022). Conversely, rapamycin administration starting at 10 weeks of age was unable to rescue the deficit (Tsai et al., 2018).

Here, we find that *Nf1* likely regulates NMJ synaptic transmission via a developmental role, since its early, embryonic, downregulation is sufficient to induce persistent deficits in the third instar larva. An early, *Nf1*-dependent developmental window has also been identified in the regulation of *Drosophila* motor activity, with *Nf1* expression required within the pupa, but not the adult, to regulate grooming behavior in the latter (King et al., 2020). In addition, the loss of function of other ASD-associated genes required for NMJ development, such as the adaptor protein *ank2* (Koch et al., 2008; Stevens and Rasband, 2021) and the presynaptic adhesion molecular *neurexin-1* (J. Li et al., 2007; Hu et al., 2019), has also been shown to disrupt third instar larval NMJ synaptic transmission. Thus, that the NF1 protein regulates synaptic transmission in a developmental manner is not necessarily surprising, although the mechanism via which it does so remains to be investigated. One possibility is via the regulation of reactive oxygen species (ROS) generation. ROS generation is an important contributor to the regulation of NMJ development, structure, and plasticity (Milton et al., 2011; Oswald et al., 2018), and excessive mitochondrial ROS levels are observed in *Nf1*^−/−^ flies, giving rise to a reduced lifespan and poor stress tolerance (Tong et al., 2007). In *Nf1^P1^* larvae, early abnormalities in ROS production may lead to abnormal NMJ development, which in turn may give rise to persistent changes in synaptic transmission.

In GABAergic hippocampal neurons of mice, loss of *Nf1* causes enhanced inhibitory transmission via increased Ras/MAPK-dependent phosphorylation of synapsin I, a synaptic vesicle protein that, on phosphorylation, dissociates from vesicles to facilitate their recruitment to the readily-releasable pool (Cui et al., 2008). Thus, the mechanism of *Nf1*-dependent transmission here appears to be physiological, rather than developmental. Yet, differences in how the loss of *Nf1* impacts neurotransmission in the mouse hippocampus relative to that at the *Drosophila* NMJ have already been discussed (Dyson et al., 2022), suggesting that *Nf1* may regulate this process via multiple mechanisms, and/or in a cell type-dependent manner. It should also be noted that, given the temporal resolution of the UAS/GAL4/GAL80^ts^ system (McGuire et al., 2004), we cannot entirely rule out an additional requirement for *Nf1* in the early first instar larval stage.

In contrast to NMJ phenotypes, constitutive loss of *Nf1* is required to induce larval tactile hypersensitivity. One possible explanation for this difference is that, while abnormal synaptic transmission may contribute to tactile hypersensitivity, other pathophysiological mechanisms, occurring within the larval stage, are also necessary, such that the former is insufficient to induce the behavioral phenotype alone. Alternatively, while *Nf1* may regulate synaptic transmission at the NMJ in a developmental manner, it may not function similarly at central synapses, as it was previously shown that tactile hypersensitivity in *Nf1^P1^* larvae likely arises from a central, cholinergic deficit (Dyson et al., 2022). It is also conceivable that *Nf1* may be required for some later compensatory mechanism that does not directly correct synaptic dysfunction but, nevertheless, ensures that it does not lead to changes in mechanosensory behavior. This may mean that, in the *Nf1^P1^* mutant, no compensation occurs following early changes to synaptic transmission because the *Nf1* gene is permanently deleted; conversely, in the UAS/GAL4/GAL80^ts^ paradigm, re-expression of *Nf1* in later larval stages following embryonic knock-down permits compensatory changes to prevent deficits in behavior. Future work is required to investigate these, and other, possibilities, and to also narrow down the *Nf1*-dependent critical period of synaptic transmission.

Regardless of the precise timings of *Nf1* function, we have identified two compounds that are able to improve tactile hypersensitivity and impaired synaptic transmission in *Nf1^P1^* larvae. These are the HMG CoA-reductase inhibitor simvastatin, and the BK_Ca_ channel activator BMS-204352. Importantly, neither drug influenced synaptic transmission in the K33 control line. Yet, it should be noted that feeding is increased in *Nf1^P1^* adult flies (Botero et al., 2021); if the same is true in *Nf1^P1^* larvae, this raises the possibility that they ingest more drug than K33 controls. Consequently, the lack of effect of simvastatin and BMS-204352 on synaptic transmission in K33 larvae may, in part, be attributed to a comparatively lower amount of compound being present, rather than indicating the efficacy of the compounds in managing *Nf1*-dependent pathophysiological mechanisms. Conversely, increased feeding in *Nf1^P1^* flies is suggested to act as a homeostatic response to an increased metabolic rate (Botero et al., 2021), which would suggest that the amount of drug active in both lines should be similar.

Simvastatin, currently prescribed as a cholesterol-lowering medication, has been examined in clinical trials for its efficacy in treating behavioral symptoms of neurofibromatosis type 1, with mixed success. It did not improve cognition in two large randomized controlled trials of children and adolescents (Krab et al., 2008; van der Vaart et al., 2013), possibly because the intervention occurred too late, with it being suggested that simvastatin treatment may have been beneficial in younger children (van der Vaart et al., 2013). Our data support this, since the improvement in tactile hypersensitivity in *Nf1^P1^* larvae was strongest when administration of the compound throughout the larval stage was combined with embryonic exposure. Indeed, some improvement in ASD symptomatology following simvastatin treatment was demonstrated in a more recent, pilot study of younger children with neurofibromatosis type 1 (Stivaros et al., 2018). The same study also concluded that simvastatin is well-tolerated in such children (Stivaros et al., 2018), in contrast to many therapies currently prescribed for co-morbid behavioral symptoms in ASD (Aishworiya et al., 2022). However, if a drug must indeed be present during early (e.g., embryonic) as well as later development to elicit an effect, as our data suggest, this may entail considerable practical and ethical implications in the clinic.

BMS-204352 currently has no clinical application, having originally been developed as a therapy for acute ischemic stroke but displaying no benefit over placebo in Phase III trials (Jensen, 2002). Since nanomolar concentrations of BMS-204352 are sufficient to activate the BK_Ca_ channel *in vitro* (Jensen, 2002), it most likely exerts its effect on sensory sensitivity via this mechanism, thereby implicating the reduced expression, or impaired activity, of BK_Ca_ channels in tactile hypersensitivity and impaired transmission in *Nf1^P1^* larvae. This is consistent with a role for BK_Ca_ channels at the plasma membrane in regulating neuronal excitability (N’Gouemo, 2014). However, BK_Ca_ channels are also expressed in mitochondria, where their activity is both important for the regulation of ROS, and susceptible to manipulation by oxidizing agents (Balderas et al., 2015; Hermann et al., 2015). Given the possible involvement of ROS in *Nf1*-dependent NMJ development and activity suggested above, the modulation of ROS may comprise an alternative mechanism by which BMS-204352 influences synaptic transmission (and behavior) via BK_Ca_.

Roles for *Nf1* in regulating potassium currents have been demonstrated previously, as the postsynaptic K^+^ current typically elicited by application of PACAP38 is diminished at the *Nf1^P1^* NMJ (Guo et al., 1997), and treatment with apamin, an inhibitor of the SK_Ca_ channel, rescues spatial learning deficits in *Nf1*^+/−^ mice (Kallarackal et al., 2013). Furthermore, haploinsufficiency of *KCNMA1* (encoding BK_Ca_) has been previously identified in an ASD individual, resulting in a diminished BK_Ca_ current in patient-derived lymphoblastoid cells that was enhanced via application of BMS-204352 (Laumonnier et al., 2006). BMS-204352 treatment has also been shown to rescue sensory hypersensitivity (Zhang et al., 2014), altered social preference (Hébert et al., 2014), and hyperactivity in an unfamiliar environment (Carreno-Munoz et al., 2018) in mouse models of the syndromic ASD Fragile X. In addition, BMS-204352 and an alternative BK_Ca_ activator, BMS-191001, were found to rescue structural deficits (Hébert et al., 2014) and hyperexcitability (Zhang et al., 2014), respectively, in dendrites. Thus, our finding may hint at a shared mechanism underlying sensory hypersensitivity, and potentially other symptoms, in different forms of syndromic ASD. To further explore this possibility, one should seek to confirm that the improvement elicited by BMS-204352 is indeed mediated via BK_Ca_, and not via other channels that the compound is also able to activate, such as those of the KCNQ family (Jensen, 2002). To do so, BMS-204352 may be administered alongside a blocker of the BK_Ca_ channel, such as paxilline or iberiotoxin, to determine whether this abrogates the effect (Balderas et al., 2015). In addition, the genetic upregulation of *slowpoke* (encoding the *Drosophila* BK_Ca_ channel α subunit) in *Nf1^P1^* larvae would, if leading to successful phenotypic rescue, strengthen the association of BK_Ca_ dysfunction with *Nf1^P1^*-dependent tactile hypersensitivity and impaired synaptic transmission.

On the other hand, there is some question over the feasibility of targeting BK_Ca_ via BMS-204352 in a clinical context. Other studies have considered the utility of BK_Ca_ as a target in the treatment of epilepsy, a neurologic disorder characterized by an excess of excitatory activity (N’Gouemo, 2011). However, both loss-of-function and gain-of-function mutations within the channel can give rise to neuronal hyperexcitability, and different anti-epileptic drugs have opposing effects on BK_Ca_ activity. Moreover, pharmacological BK_Ca_ activation may either protect against or, conversely, induce seizures in different contexts. As such, the effective modulation of BK_Ca_ channels as a treatment for neurodevelopmental disorders may be challenging (N’Gouemo, 2011, 2014). Although Phase I and II trials have demonstrated that BMS-204352 is safe and well-tolerated in both healthy adults and acute stroke patients (Jensen, 2002), whether this will also be the case in young children, and over an extended dosing period, is unknown.

Despite demonstrating the consistent efficacy of simvastatin and BMS-204352 in improving *Nf1*-dependent tactile hypersensitivity in four subsequent trials, we were unable to fully rescue the phenotype, since K33 control larvae typically show a 0–20% likelihood of responding in the mechanoreception assay (Dyson et al., 2022). One explanation is that we did not test compounds at optimum concentrations. Alternatively, it may be because the target of the drug is not the only mechanism concerned. For instance, while BK_Ca_ dysfunction may contribute to tactile hypersensitivity, the dysfunction of other ion channels, for example, may also be involved, which must also be pharmacologically corrected to completely rescue the phenotype. However, it is difficult to apply this reasoning to simvastatin: although we have not shown biochemically that simvastatin functions in our assay by targeting Ras (e.g., via Western blot of larval CNS extracts to examine phospho-MAPK levels following treatment), the most parsimonious explanation for its effect is that, by inhibiting HMG CoA-reductase, it prevents the farnesylation of Ras and its subsequent anchoring to the plasma membrane (W. Li et al., 2005). Yet, genetically attenuating Ras protein expression via RNA interference fully rescues the hypersensitivity phenotype (Dyson et al., 2022).

We did not examine the possibility of a combinatorial effect of the compounds on tactile hypersensitivity, such that administering both simvastatin and BMS-204352 simultaneously results in a stronger phenotypic rescue. Yet, if the target of BMS-204352 (assumed to be altered BK_Ca_ activity) arises directly downstream of elevated Ras/MAPK signaling, the utility of combinatorial therapy here may be limited. Alternatively, combining simvastatin or BMS-204352 with compounds targeting distinct molecular pathways may provide a more effective approach. Such compounds may include antioxidant therapies, like N-acetylcysteine, which was found to improve irritability and stereotypy in children with ASD (Hardan et al., 2012), and may function in part by ameliorating an excitation/inhibition imbalance via a reduction in glutamatergic transmission (Dean et al., 2011; Hardan et al., 2012). Oxidative stress has previously been implicated in the pathophysiology of idiopathic ASD (Bjørklund et al., 2020), and the possibility of abnormal ROS underlying defective transmission in *Nf1*^−/−^ larvae has been considered above. Screening antioxidant compounds in such animals may shed light on this proposed mechanism.

A subsequent screen may also involve activators of the cAMP/PKA pathway. The NF1 protein acts as a positive regulator of cAMP/PKA signaling by stimulating adenylyl cyclase activity (Guo et al., 2000; Tong et al., 2002), and genetic or pharmacological stimulation of this pathway rescues anatomic and behavioral phenotypes in both fly and mouse models of neurofibromatosis type 1 (Guo et al., 2000; Tsai et al., 2012; Diggs-Andrews et al., 2013; Sutton et al., 2019). While knock-down of Ras expression is sufficient to rescue tactile hypersensitivity and abnormal synaptic transmission in *Nf1^P1^* larvae (Dyson et al., 2022), this does not necessarily rule out impaired cAMP/PKA signaling in the mechanism, since the NF1 protein is capable of regulating both Ras and cAMP even within the same pathway(s) (Anastasaki and Gutmann, 2014; Walker and Bernards, 2014).

To translate our findings into the clinic, it will be important to test these compounds in mammalian models of neurofibromatosis type 1 and/or other syndromic forms of ASD. Unlike *Drosophila* larvae, rodent models of neurofibromatosis type 1 do not consistently and strongly exhibit enhanced sensitivity to a mechanical stimulus, and while such models may display thermal hyperalgesia, this is considered a proxy for chronic pain in neurofibromatosis type 1 and arises because of sensory impairment (Moutal et al., 2017; Bellampalli and Khanna, 2019). Conversely, tactile hypersensitivity in *Nf1*^−/−^ larvae likely originates in the CNS, and may therefore have different mechanistic underpinnings (Dyson et al., 2022). Nevertheless, *Nf1*^+/−^ mice do display behaviors reflecting other aspects of ASD symptomatology, such as altered social behavior and communication (Molosh et al., 2014; Maloney et al., 2018). Since a single compound may not target multiple ASD symptom domains, before preclinical testing, it would be useful to test simvastatin and BMS-204352 on phenotypes in the fly that are also analogous to social impairment, such as defective courtship (Moscato et al., 2020). Additionally, determination of the efficacy of these compounds on their purported mechanistic targets, e.g., MAPK phosphorylation and BK_Ca_ currents, respectively, in *Nf1*^+/−^ cell lines derived from human patients may prove fruitful.

In summary, we have shed light on the temporal requirements for the NF1 protein in regulating two previously identified larval phenotypes that arguably parallel ASD symptomatology and etiology. Subsequently, we identified two compounds that improve, but do not necessarily fully rescue, the same phenotypes. Despite the previous failure of simvastatin to improve cognitive symptoms in children with neurofibromatosis type 1 (Krab et al., 2008; van der Vaart et al., 2013), our findings suggest that the further investigation of this compound, and of BMS-204352, specifically in the context of ASD may be warranted.

## Synthesis

Reviewing Editor: Lynda Erskine, University of Aberdeen Decisions are customarily a result of the Reviewing Editor and the peer reviewers coming together and discussing their recommendations until a consensus is reached. When revisions are invited, a fact-based synthesis statement explaining their decision and outlining what is needed to prepare a revision will be listed below. The following reviewer(s) agreed to reveal their identity: Seth Tomchik, Claudio Alonso.

Your manuscript has been reviewed by 2 experts in the field. As you will see below, they are both agreed that these are important findings supported by strong data. However, they have some suggestions, largely textural, for improving the manuscript. Below is a synthesis of the reviews, listing the points that should be addressed prior to publication.

General comments from reviewers:

This manuscript describes a low-throughput screen to identify pharmacological modifiers of NF1 phenotypes at the Drosophila NMJ. The report details the developmental/temporal requirements for Nf1 for the NMJ synaptic transmission and tactile sensitivity, and further identifies two drugs that partially rescue the tactile sensitivity phenotype. One targets a known Nf1 interacting pathway, while the other represents a novel target for potential future development. The data look good overall and the manuscript is well written.

This study uses a Drosophila model to identify modulators of the symptomatology linked to autism spectrum disorder (ASD). In particular, the authors use a specific Drosophila line that lacks normal expression of the fruit fly’s orthologue of the Neurofibromatosis Type 1 (NF1) gene, which shows a sensory phenotype reminiscent of ASD, and conduct a search for compounds that might improve/rescue the NF1 phenotype in regards to tactile sensitivity finding two interesting compounds termed simvastin and BMS-204352. Given that therapeutic approaches to ASD are limited (behavioural interventions remain the dominant treatment) the work has potential to advance an important area of research with the prospects of impact on the design of clinical therapies for ASD in the future. The paper is of high quality and fits well the remit of eNeuro regarding contributions towards the understanding of disorders of the nervous system.

Major points to be addressed:

1. The authors claim there are no pharmacological therapies that effectively target ASD core symptomatology but this is an overstatement and should be corrected (see for example Aishworiya et al. 2022). The authors should describe the full range of pharmacological approaches currently available to treat ASD and some of their side-effects, advantages/disadvantages.

Additionally, in the Discussion, the authors should use the existing ASD pharmacological framework to describe their findings, comparing the potential benefits/limitations of the compounds identified with other pharmacological approaches available.

2. The authors should explain in more detail the rationale for selecting compounds for testing. They do explain that they aimed at factors with impact on Ras/MAPK signalling -and their justification seems reasonable- but it is unclear what other pathways or functions would have also been good candidates for a chemical screen. This will help the reader to put the new findings in perspective.

3. It would be helpful if a series of videos of the (already conducted) experiments is added to the manuscript to provide the reader with more information about the tests carried out.

4. Sample sizes in Fig. 1 and Fig. 4 A-F are very low. While these are individual population assays, running only n=1-4 is likely underpowered.

5. Regarding the lack of a full phenotypic rescue after either simvastin or BMS-204352 treatment: have the authors considered the possibility that the compounds identified might have combinatorial/synergic effects on the system? Could they test this notion and consider a pilot “double treatment” with simvastin and BMS-204352 to determine additive/non-additive effects?

6. There is some question about the potential therapeutic utility of targeting BKCa BMS-204352 in treating NF1 cognitive/behavioral symptoms in humans. More discussion about how manipulation of this type of channel might work in humans (in comparison with what is known about similar approaches re: other channels/disorders) would be beneficial for the manuscript. For instance, is there evidence that manipulation of similar channels is an effective intervention for any other disorder with ASD-like symptomology? Is administration of BMS-204352 theoretically viable over relatively long time periods (given that it was developed/tested for ischemia)?

7. The authors should expand their discussion of the mechanisms by which the identified compounds simvastin and BMS-204352 might impact sensory function, suggesting experimental routes to test such models in the future.

8. The data here suggest that there is an early developmental plasticity window for Nf1 in regulating NMJ development, which the authors compare/contrast with the potential for developmental contributions to the rodent spatial learning phenotype. It is also noteworthy (and more relevant) that, in Drosophila, there is an Nf1-sensitive developmental window during pupal development that supports the development of normal motor activity patterns (grooming).

9. There are alterations in metabolic rate, feeding, ROS production / oxidative stress, and mitochondrial function in Drosophila nf1 mutants (Tong et al., 2007; Botero et al., 2022). Could this affect the observed phenotypes at the NMJ? Consideration of metabolic interactions - both with drug metabolism and NMJ phenotypes - would place the present findings into a larger context. There is an increase in feeding in the nf1P1 mutants/RNAi (Botero et al., 2022), which would be expected to increase drug intake in those groups (assuming equivalence in larva and no systematic difference in drug taste perception between genotypes) - this should be noted/discussed.

10. Based on their results, are the authors able to suggest further targeted screens? If yes/no could they explain why, and add these elements to the discussion.

11. The authors should discuss the steps required to bridge their findings with a potential clinical pathway for ASD treatment.

Additional points:

Double-check the consistency of the nomenclature and abbreviations (e.g., capitalization of Nf1P1, BMS-204352 vs. -204532). The disease name neurofibromatosis type 1 is not typically capitalized, but either way, should be consistent across the abstract/main text, etc.

## Author Response

Major Point 1

“The authors claim there are no pharmacological therapies that effectively target ASD core symptomatology but this is an overstatement and should be corrected (see for example Aishworiya et al. 2022). The authors should describe the full range of pharmacological approaches currently available to treat ASD and some of their side-effects, advantages/disadvantages. Additionally, in the Discussion, the authors should use the existing ASD pharmacological framework to describe their findings, comparing the potential benefits/limitations of the compounds identified with other pharmacological approaches available.”

The Introduction has been rewritten to include the following passage, in which we refer to current treatments for comorbid behavioral issues in ASD, the adverse effects that may be associated with them, and the fact that they have not been shown to consistently improve core ASD symptomatology:

“The need for such interventions is urgent, given the lifelong impact on an individual’s quality of life that ASD can impose (Lord et al., 2020), and the substantial economic burden arising from the need to support those affected (Buescher et al., 2014; Leigh and Du, 2015). Currently, the only two compounds approved by the FDA for the treatment of behavioral symptoms in ASD are the atypical antipsychotics aripiprazole and risperidone, both of which are prescribed for irritability and aggression (Lord et al., 2020). Additional medications commonly prescribed to individuals with ASD include stimulant drugs for the treatment of comorbid ADHD, selective serotonin-reuptake inhibitors for mood disturbances and anxiety, and melatonin for sleep disorders (Aishworiya et al., 2022). However, these are often associated with adverse side effects ranging from the mild (e.g. weight gain, appetite changes, fatigue, and headaches) to the severe (e.g. metabolic syndrome) (Aishworiya et al., 2022). Importantly, none have been shown to consistently and effectively improve abnormal sensory sensitivity or indeed other aspects of core ASD symptomatology (Hyman et al., 2020).”

In the Discussion, the safety of simvastatin in young children is acknowledged (and contrasted with the adverse effects common amongst existing pharmacotherapies), with the following statement:

“The same study also concluded that simvastatin is well-tolerated in such children (Stivaros et al., 2018), in contrast to many therapies currently prescribed for co-morbid behavioral symptoms in ASD (Aishworiya et al., 2022).”

Furthermore, we now state in the Discussion that BMS-204352 appears safe in healthy adults, but that it is unknown whether this is also the case in children, and over an extended period:

“Although phase I and II trials have demonstrated that BMS-204352 is safe and well-tolerated in both healthy adults and acute stroke patients (Jensen, 2022), whether this will also be the case in young children, and over an extended dosing period, is unknown.”

2

Major Point 2

“The authors should explain in more detail the rationale for selecting compounds for testing. They do explain that they aimed at factors with impact on Ras/MAPK signalling -and their justification seems reasonable- but it is unclear what other pathways or functions would have also been good candidates for a chemical screen. This will help the reader to put the new findings in perspective.”

In the Results section, we have expanded on the reasons for focusing on Ras/MAPK pathway inhibitors and ion channel modulators:

“Tested compounds fell broadly into one of two classes of activity (Table 1). First, compounds were selected for their ability to diminish Ras/MAPK signaling, since the NF1 protein acts as a negative regulator of Ras (Ratner and Miller, 2015), knockdown of either Ras85D or Ras64B fully rescues both tactile hypersensitivity and synaptic dysfunction in Nf1P1 larvae (Dyson et al., 2022), and genetic variants associated with Ras/MAPK pathways are recurrent in idiopathic ASD (Luo et al., 2018). The second class comprised compounds known to modulate ion channel function, typically in favor of reducing neuronal excitability, as synaptic transmission deficits associated with tactile hypersensitivity in Nf1P1 larvae are indicative of neuronal hyperexcitability (Dyson et al., 2022), and an imbalance in central excitatory/inhibitory activity has been proposed as a major pathophysiological mechanism underlying ASD (Rubenstein and Merzenich, 2003).”

In addition, towards the end of the Discussion, we make suggestions for alternative compound classes to be screened (i.e. antioxidant therapies and cAMP/PKA modulators):

”...Such compounds may include antioxidant therapies, like N-acetylcysteine, which was found to improve irritability and stereotypy in children with ASD (Hardan et al., 2012), and may function in part by ameliorating an excitation/inhibition imbalance via a reduction in glutamatergic transmission (Dean et al., 2011; Hardan et al., 2012). Oxidative stress has previously been implicated in the pathophysiology of idiopathic ASD (Bjørklund et al., 2020), and the possibility of abnormal ROS underlying defective transmission in Nf1-/- larvae has been considered above. Screening antioxidant compounds in such animals may shed light on this proposed mechanism.

A subsequent screen may also involve activators of the cAMP/PKA pathway. The NF1 protein acts as a positive regulator of cAMP/PKA signaling by stimulating adenylyl cyclase activity (Guo et al., 2000; Tong et al., 2002), and genetic or pharmacological stimulation of this pathway rescues anatomical and behavioral phenotypes in both fly and mouse models of neurofibromatosis type 1 (Guo et al., 2000; Tsai et al., 2012; Diggs-Andrews et al., 2013; Sutton et al., 2019). While knockdown of Ras expression is sufficient to rescue tactile hypersensitivity and abnormal synaptic transmission in Nf1P1 larvae (Dyson et al., 2022), this does not necessarily rule out impaired cAMP/PKA signaling in the mechanism, since the NF1 protein is capable of regulating both Ras and cAMP even within the same pathway(s) (Anastasaki and Gutmann, 2014; Walker and Bernards, 2014).”

Major Point 3

“It would be helpful if a series of videos of the (already conducted) experiments is added to the manuscript to provide the reader with more information about the tests carried out.”

In the Methods section, we now refer to the videos published in Dyson et al., 2022, demonstrating the typical responses of K33 and Nf1P1 larvae:

“Video footage exemplifying the typical response of Nf1P1 larvae (representative of larvae lacking Nf1 expression) compared to that of K33 controls has already been published (Dyson et al., 2022).”

3

Major Point 4

“Sample sizes in Fig. 1 and Fig. 4 A-F are very low. While these are individual population assays, running only n=1-4 is likely underpowered.” In Figure 1, sample sizes range from n = 47 to n = 95. This falls within the range used by previous studies on mechanoreception/nociception in Drosophila larvae, e.g.:

1. Kim et al., 2012, “The role of Drosophila Piezo in mechanical nociception”, Figure 1B: For a similar nociception assay, “n = 40 [larvae] from four independent experiments.”

2. Zhou et al., 2012, “Control of directional change after mechanical stimulation in Drosophila,” Figure 2A: For a similar nociception assay, total n per genotype ranges from 74 - 91, divided into three separate trials.

3. Mauthner et al., 2014, “Balboa Binds to Pickpocket In Vivo and Is Required for Mechanical Nociception in Drosophila Larvae,” Figure 1D: For a similar nociception assay, n ranges from 62 - 67 between genotypes.

Furthermore, for the experiments in which the n value for one of the genotypes was at the lower end of this range (see GFPRNAi or K33 in panels 1A, E, F), comparisons between groups were nevertheless significant at p<0.0001. Thus, it is highly unlikely that increasing sample size will influence these results.

In Figure 4A-F, each data point represents the % responders from a single trial of 20 larvae. As there are four trials per condition, n = 80 larvae overall. Thus, the overall n falls within the range of sample sizes for nociceptive assays previously published (see list above).

In addition, the first study to demonstrate tactile hypersensitivity in Nf1-/- larvae used a similar approach (4 trials of n=20), which was sufficient to observe a consistent effect size (Dyson et al., 2022). Therefore, we contend that our sample sizes are sufficient for all experiments.

That “n = 80 overall” is now stated explicitly within the Methods.

Major Point 5

“Regarding the lack of a full phenotypic rescue after either simvastin or BMS-204352 treatment: have the authors considered the possibility that the compounds identified might have combinatorial/synergic effects on the system? Could they test this notion and consider a pilot “double treatment” with simvastin and BMS-204352 to determine additive/non-additive effects?”

We have not tested this possibility, but now consider the potential usefulness of the approach within the Discussion:

“We did not examine the possibility of a combinatorial effect of the compounds on tactile hypersensitivity, such that administering both simvastatin and BMS-204352 simultaneously results in a stronger phenotypic rescue. Yet, if the target of BMS-204352 (assumed to be altered BKCa activity) arises directly downstream of elevated Ras/MAPK signaling, the utility of combinatorial therapy here may be limited. Alternatively, combining simvastatin or BMS-204352 with compounds targeting distinct molecular pathways may provide a more effective approach.”

Major Point 6

4

“There is some question about the potential therapeutic utility of targeting BKCa BMS-204352 in treating NF1 cognitive/behavioral symptoms in humans. More discussion about how manipulation of this type of channel might work in humans (in comparison with what is known about similar approaches re: other channels/disorders) would be beneficial for the manuscript. For instance, is there evidence that manipulation of similar channels is an effective intervention for any other disorder with ASD-like symptomology? Is administration of BMS-204352 theoretically viable over relatively long time periods (given that it was developed/tested for ischemia)?” The concerns regarding the therapeutic utility of a) targeting BMS-204352, and b) doing so via BMS-204352, in the context of neurodevelopmental disorders, are acknowledged in the Discussion:

“On the other hand, there is some question over the feasibility of targeting BKCa via BMS-204352 in a clinical context. Other studies have considered the utility of BKCa as a target in the treatment of epilepsy, a neurological disorder characterized by an excess of excitatory activity (N’Gouemo, 2011). However, both loss- and gain-of-function mutations within the channel can give rise to neuronal hyperexcitability, and different anti-epileptic drugs have opposing effects on BKCa activity. Moreover, pharmacological BKCa activation may either protect against or, conversely, induce seizures in different contexts. As such, the effective modulation of BKCa channels as a treatment for neurodevelopmental disorders may be challenging (N’Gouemo, 2011; N’Gouemo, 2014). Although phase I and II trials have demonstrated that BMS-204352 is safe and well-tolerated in both healthy adults and acute stroke patients (Jensen, 2022), whether this will also be the case in young children, and over an extended dosing period, is unknown.”

Major Point 7

“The authors should expand their discussion of the mechanisms by which the identified compounds simvastin and BMS-204352 might impact sensory function, suggesting experimental routes to test such models in the future.”

In the Discussion, we suggest an experiment to test our suggestion that simvastatin elicits an effect by diminishing Ras activity:

”...(e.g., via Western blot of larval CNS extracts to examine phospho-MAPK levels following treatment)...”

We also explain that BMS-204352 is assumed to work via BKCa because:

”...nanomolar concentrations of BMS-204352 are sufficient to activate the BKCa channel in vitro (Jensen, 2002)...”

Furthermore, we acknowledge that BMS-204352 may have other targets, and suggest ways to demonstrate that it does indeed work through BKCa:

”...one should seek to confirm that the improvement elicited by BMS-204352 is indeed mediated via BKCa, and not via other channels that the compound is also able to activate, such as those of the KCNQ family (Jensen, 2002). To do so, BMS-204352 may be administered alongside a blocker of the BKCa channel, such as paxilline or iberiotoxin, to determine whether this abrogates the effect (Balderas et al., 2015). In addition, the genetic upregulation of slowpoke (encoding the Drosophila BKCa channel alpha subunit) in Nf1P1 larvae would, if leading to successful phenotypic rescue, strengthen the association of BKCa dysfunction with Nf1P1-dependent tactile hypersensitivity and impaired synaptic transmission.”

Major Point 8

“The data here suggest that there is an early developmental plasticity window for Nf1 in regulating NMJ development, which the authors compare/contrast with the potential 5 for developmental contributions to the rodent spatial learning phenotype. It is also noteworthy (and more relevant) that, in Drosophila, there is an Nf1-sensitive developmental window during pupal development that supports the development of normal motor activity patterns (grooming).”

We now refer to the Nf1-sensitive developmental window for grooming in the

Discussion:

“An early, Nf1-dependent developmental window has also been identified in the regulation of Drosophila motor activity, with Nf1 expression required within the pupa, but not the adult, to regulate grooming behavior in the latter (King et al., 2020).”

Major Point 9

“There are alterations in metabolic rate, feeding, ROS production / oxidative stress, and mitochondrial function in Drosophila nf1 mutants (Tong et al., 2007; Botero et al., 2022). Could this affect the observed phenotypes at the NMJ? Consideration of metabolic interactions - both with drug metabolism and NMJ phenotypes - would place the present findings into a larger context. There is an increase in feeding in the nf1P1 mutants/RNAi (Botero et al., 2022), which would be expected to increase drug intake in those groups (assuming equivalence in larva and no systematic difference in drug taste perception between genotypes) - this should be noted/discussed.”

In the Discussion, we consider the possibility that aberrant ROS may contribute to NMJ dysfunction:

“ROS generation is an important contributor to the regulation of NMJ development, structure, and plasticity (Milton et al., 2011; Oswald et al., 2018), and excessive mitochondrial ROS levels are observed in Nf1-/- flies, giving rise to a reduced lifespan and poor stress tolerance (Tong et al., 2007). In Nf1P1 larvae, early abnormalities in ROS production may lead to abnormal NMJ development, which in turn may give rise to persistent changes in synaptic transmission.”

We also acknowledge that BMS-204532 may influence ROS via BKCa:

“BKCa channels are also expressed in mitochondria, where their activity is both important for the regulation of ROS, and susceptible to manipulation by oxidizing agents (Balderas et al., 2015; Hermann et al., 2015). Given the possible involvement of ROS in Nf1-dependent NMJ development and activity suggested above, the modulation of ROS may comprise an alternative mechanism by which BMS-204352 influences synaptic transmission (and behavior) via BKCa.”

That differences in feeding between K33 and Nf1P1 larvae may influence drug intake, and the possibility of this influencing our results, is also noted:

“Importantly, neither drug influenced synaptic transmission in the K33 control line. Yet, it should be noted that feeding is increased in Nf1P1 adult flies (Botero et al., 2021); if the same is true in Nf1P1 larvae, this raises the possibility that they ingest more drug than K33 controls. Consequently, the lack of effect of simvastatin and BMS-204352 on synaptic transmission in K33 larvae may, in part, be attributed to a comparatively lower amount of compound being present, rather than indicating the efficacy of the compounds in managing Nf1-dependent pathophysiological mechanisms. Conversely, increased feeding in Nf1P1 flies is suggested to act as a homeostatic response to an increased metabolic rate (Botero et al., 2021), which would suggest that the amount of drug active in both lines should be similar.”

Major Point 10

“Based on their results, are the authors able to suggest further targeted screens? If yes/no could they explain why, and add these elements to the discussion.”

6

In the Discussion, we suggest that a) antioxidant therapies and b) cAMP/PKA modulators may also be useful classes of compounds to screen in a Drosophila model of neurofibromatosis type 1. For specifics, see our response to Major Point 2.

Major Point 11

“The authors should discuss the steps required to bridge their findings with a potential clinical pathway for ASD treatment.”

We offer considerations for testing these compounds in mammalian models of neurofibromatosis type 1 (as would typically be required prior to clinical trials), and suggest that they may also be examined in Nf1+/- cells derived from human patients:

“To translate our findings into the clinic, it will be important to test these compounds in mammalian models of neurofibromatosis type 1 and/or other syndromic forms of ASD. Unlike Drosophila larvae, rodent models of neurofibromatosis type 1 do not consistently and strongly exhibit enhanced sensitivity to a mechanical stimulus, and while such models may display thermal hyperalgesia, this is considered a proxy for chronic pain in neurofibromatosis type 1 and arises due to sensory impairment (Moutal et al., 2017; Bellampalli and Khanna, 2019). Conversely, tactile hypersensitivity in Nf1-/- larvae likely originates in the CNS, and may therefore have different mechanistic underpinnings (Dyson et al., 2022). Nevertheless, Nf1+/- mice do display behaviors reflecting other aspects of ASD symptomatology, such as altered social behavior and communication (Molosh et al., 2014; Maloney et al., 2018). Since a single compound may not target multiple ASD symptom domains, prior to pre-clinical testing, it would be useful to test simvastatin and BMS-204352 on phenotypes in the fly that are also analogous to social impairment, such as defective courtship (Moscato et al., 2020). Additionally, determination of the efficacy of these compounds on their purported mechanistic targets - e.g. MAPK phosphorylation and BKCa currents, respectively - in Nf1+/- cell lines derived from human patients may prove fruitful.”

Additional Points

“Double-check the consistency of the nomenclature and abbreviations (e.g., capitalization of Nf1P1, BMS-204352 vs. -204532). The disease name neurofibromatosis type 1 is not typically capitalized, but either way, should be consistent across the abstract/main text, etc.” To the best of our knowledge, the Nf1 gene (and its alleles/transgenic constructs, e.g. Nf1P1 and Nf1RNAi) is stated throughout the manuscript with a capital ‘N’ and lowercase

‘f’, aside from when referring explicitly to the human NF1 gene, as in the following sentences:

“One such condition is neurofibromatosis type 1, an autosomal dominant disorder arising from loss of function of the NF1 gene on chromosome 17 (Gutmann et al., 2017).”

“Drosophila express a highly conserved ortholog of NF1 (The et al., 1997) with similar molecular, cellular, and behavioral functions to its mammalian counterpart (Guo et al., 2000; Walker et al., 2006).”

This stylization of non-human Nf1 has also been corrected in the figures, to make it consistent with the text.

We maintain full capitalization of NF1 when mentioning the ‘NF1 protein’.

We have corrected typographical errors in which BMS-204352 was written incorrectly.

The disorder ‘neurofibromatosis type 1’ is now written consistently in lowercase.
